# Psoas muscle area is an independent survival prognosticator in patients undergoing surgery for long‐bone metastases

**DOI:** 10.1002/cam4.7072

**Published:** 2024-03-08

**Authors:** Chia‐Che Lee, Ting‐En Tseng, Ruey‐Feng Chang, Hung‐Kuan Yen, Yu‐An Chen, Yu‐Yung Chen, Chih‐Horng Wu, Ming‐Hsiao Hu, Mao‐Hsu Yen, Michiel Bongers, Olivier Q. Groot, Cheng‐Yo Lai, Wei‐Hsin Lin

**Affiliations:** ^1^ Graduate Institute of Biomedical Electronics and Bioinformatics National Taiwan University Taipei Taiwan; ^2^ Department of Orthopaedic Surgery National Taiwan University Hospital Taipei Taiwan; ^3^ Department of Orthopaedic Surgery National Taiwan University Hospital Hsinchu Taiwan; ^4^ Department of Medical Education National Taiwan University Hospital Hsinchu Taiwan; ^5^ Department of Medical Education National Taiwan University Hospital Taipei Taiwan; ^6^ Department of Medical Imaging National Taiwan University Hospital Taipei Taiwan; ^7^ Department of Computer Science and Engineering National Taiwan Ocean University Keelung Taiwan; ^8^ Department of Orthopaedic Surgery Massachusetts General Hospital Boston Massachusetts USA; ^9^ Department of Orthopaedics University Medical Center Utrecht Utrecht The Netherlands

**Keywords:** Asian cohort, extremity metastasis, psoas muscle area, survival prediction

## Abstract

**Background:**

Predictive analytics is gaining popularity as an aid to treatment planning for patients with bone metastases, whose expected survival should be considered. Decreased psoas muscle area (PMA), a morphometric indicator of suboptimal nutritional status, has been associated with mortality in various cancers, but never been integrated into current survival prediction algorithms (SPA) for patients with skeletal metastases. This study investigates whether decreased PMA predicts worse survival in patients with extremity metastases and whether incorporating PMA into three modern SPAs (PATHFx, SORG‐NG, and SORG‐MLA) improves their performance.

**Methods:**

One hundred eighty‐five patients surgically treated for long‐bone metastases between 2014 and 2019 were divided into three PMA tertiles (small, medium, and large) based on their psoas size on CT. Kaplan–Meier, multivariable regression, and Cox proportional hazards analyses were employed to compare survival between tertiles and examine factors associated with mortality. Logistic regression analysis was used to assess whether incorporating adjusted PMA values enhanced the three SPAs' discriminatory abilities. The clinical utility of incorporating PMA into these SPAs was evaluated by decision curve analysis (DCA).

**Results:**

Patients with small PMA had worse 90‐day and 1‐year survival after surgery (log‐rank test *p* < 0.001). Patients in the large PMA group had a higher chance of surviving 90 days (odds ratio, OR, 3.72, *p* = 0.02) and 1 year than those in the small PMA group (OR 3.28, *p* = 0.004). All three SPAs had increased AUC after incorporation of adjusted PMA. DCA indicated increased net benefits at threshold probabilities >0.5 after the addition of adjusted PMA to these SPAs.

**Conclusions:**

Decreased PMA on CT is associated with worse survival in surgically treated patients with extremity metastases, even after controlling for three contemporary SPAs. Physicians should consider the additional prognostic value of PMA on survival in patients undergoing consideration for operative management due to extremity metastases.

## 1 INTRODUCTION

Long‐bone metastases are common in patients with advanced cancer and, if not properly managed, could result in a substantial reduction in patients' quality of life.^1^, ^2^ Survival estimation is important when clinicians contemplate treatment options for an impending or actual pathological fracture.^3^ Patients with a longer life expectancy are more likely to benefit from extensive surgeries. In contrast, patients with a short life expectancy are better managed with non‐operative treatment or less invasive procedures aimed at improving their quality of life^2^, ^3^, ^4^

Several survival prediction algorithms (SPAs), such as the Scandinavian Sarcoma Group (SSG) scale, OPTIModel, metastatic early prognostic (MEP) score, SORG nomogram (SORG‐NG), and SPRING nomogram (SPRING‐NG), were created to help clinicians make survival predictions in patients with skeletal metastases.^5^, ^6^, ^7^, ^8^, ^9^ More recently, two machine learning‐based systems were introduced: PATHFx and the Skeletal Oncology Research Group Machine Learning Algorithm (SORG‐MLA). PATHFx, a Bayesian belief network‐based algorithm developed in 2011, has been validated in multiple regions such as the United States, Japan, Italy, the Scandinavian peninsula, and Taiwan.^10^ It is currently in its third iteration as an online application for clinical use.^11^ SORG‐MLA was introduced in 2020 as a web‐based application that incorporated 15 prognostic factors and has been shown to achieve great discrimination on both internal and external validations.^12^, ^13^, ^14^ All of the SPAs mentioned above, whether machine learning‐based or not, included prognostic factors limited to demographic, clinical, oncological, and laboratory data.^5^, ^6^, ^7^, ^8^, ^9^, ^11^, ^12^, ^13^, ^14^, ^15^ None of them directly takes body composition factors such as psoas muscle area (PMA) into consideration. Since PATHFx, SORG‐NG, and SORG‐MLA have demonstrated excellent discriminatory ability in their development studies and have been extensively validated,^13^, ^14^, ^15^ we focused on evaluating the performance of these three models.

Computed tomography (CT) is often used to monitor visceral tumors, can be acquired quickly, and often is well tolerated by immobile patients. Psoas muscle area at the L3 vertebra measured on CT images has been shown to be a predictor of mortality in patients with cancer.^16^, ^17^, ^18^, ^19^, ^20^, ^21^, ^22^, ^23^ If body measurements such as PMA provide additional predictive value independent of the existing SPAs, incorporating morphometric factors into these tools could potentially improve their performance and help patients and clinicians make better‐informed decisions. In this study, we would like to ask (1) whether decreased PMA on CT is associated with worse 90‐day, 1‐year, and overall survival in patients with extremity metastases? And (2) whether incorporating PMA into the three state‐of‐the‐art SPAs improves their model performance?

## 2 MATERIALS AND METHODS

### 2.1 Ethics statement

This study followed the Declaration of Helsinki and the STROBE guidelines.^24^ This study was approved by our research ethics committee (201912022RIND). Informed consent was waived due to its retrospective nature.

### 2.2 Study design and participants

**FIGURE 1:**
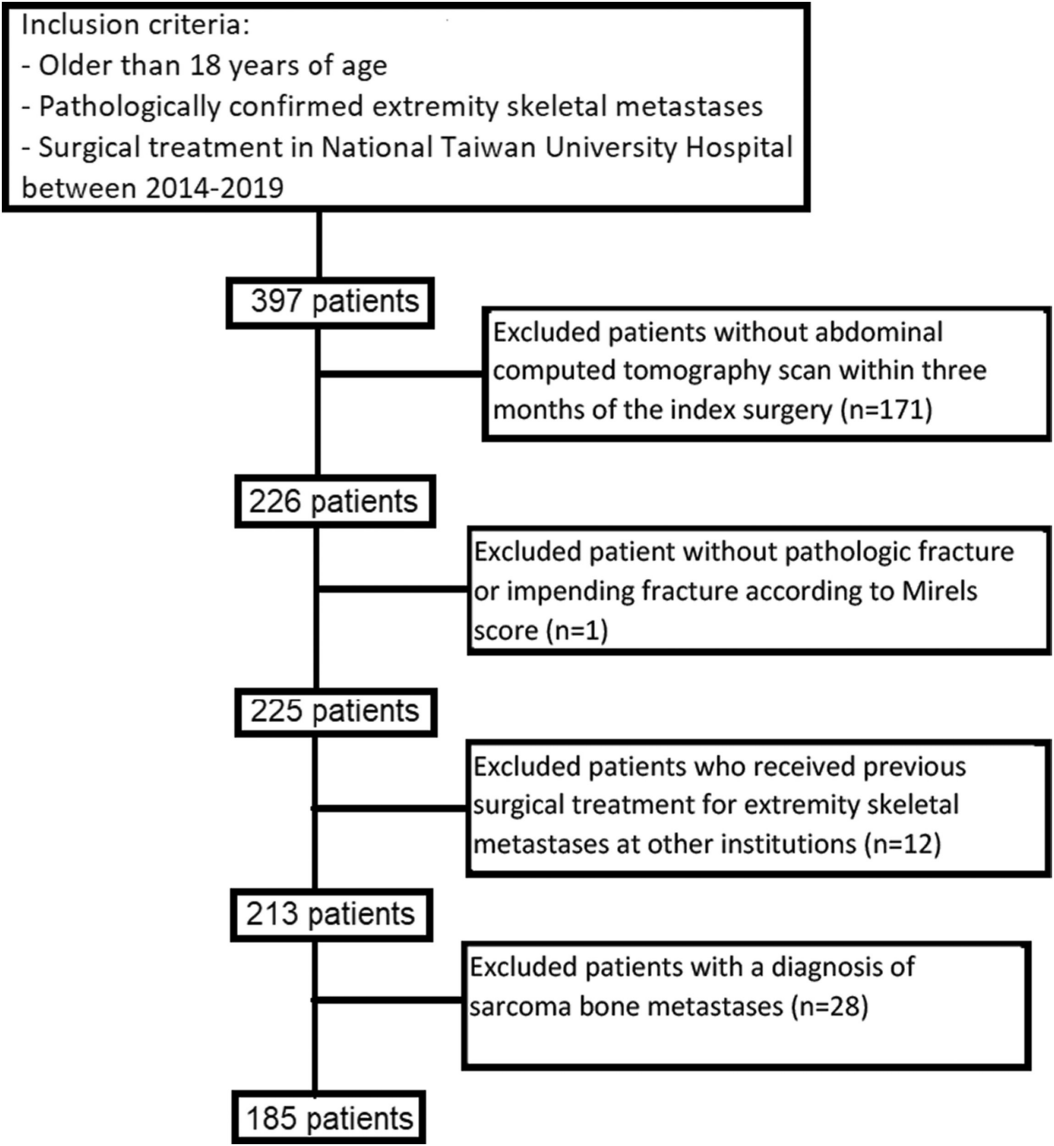
Flowchart of the study's inclusion criteria.

This retrospective study included 185 patients undergoing surgical treatment for a long‐bone metastasis in the upper or lower extremities at a single tertiary medical center in Taiwan between 2014 and 2019. The surgical decision was made based on a multi‐disciplinary discussion among the surgeon, medical oncologist, and radiation oncologist, and after discussion with the patients. Included patients met the following criteria: (1) 18 years of age or older; (2) histologically confirmed skeletal metastasis in an extremity from visceral cancer other than sarcoma on the pathology report of surgical specimen(s); and (3) the nearest computed tomography (CT) scan was within 3 months of the index surgery and encompassed the psoas muscles (Figure 1).

### 2.3 Survival prediction algorithms

In the literature, PATHFx, SORG‐NG, and SORG‐MLA demonstrated excellent discriminatory ability in their development studies and have been externally validated multiple times.^13^, ^14^, ^15^ We therefore focused on examining whether incorporating PMA improved the performance of these three models. 90 days and 1 year were the only survival periods included in all three SPAs. Thus, we compared performance between models at these two timepoints.

### 2.4 Outcome and clinical variables

The primary outcomes were survival at 90 days and 1 year after the index surgery, with the endpoint being death by any cause. To eliminate the potential influence from outliers, survival data were censored at postoperative 2 years. Loss to follow‐up was 1% (1/185) at 90 days and 11% (20/185) at 1 year.

The following variables were acquired for the three SPAs: gender; age; height; weight; body mass index; preoperative American Society of Anesthesiologists (ASA) physical status classification; preoperative Eastern Cooperative Oncology Group (ECOG) performance status; preoperative Charlson Comorbidity Index; preoperative ambulatory status; whether preoperative systemic therapy or preoperative radiotherapy was given; the number of bone metastases; the presence of brain or visceral metastasis; primary tumor type; and 12 preoperative laboratory values including hemoglobin level; white blood cell count; platelet count; absolute lymphocyte count; absolute neutrophil count; serum creatinine level; serum albumin level; serum alkaline phosphatase level; serum sodium level; serum calcium level; serum blood urea nitrogen level; and international normalized ratio. All variables were recorded in compliance with their original definitions provided in the corresponding SPA development studies.

### 2.5 CT variables

Two well‐trained raters, including a radiologist with more than 10 years of experience in abdominal imaging, performed morphometric analysis of the psoas muscle area and the spine body area at the L3 vertebral level. The methods used to outline and measure these two areas were described in previous publications.^18^, ^20^

**FIGURE 2:**
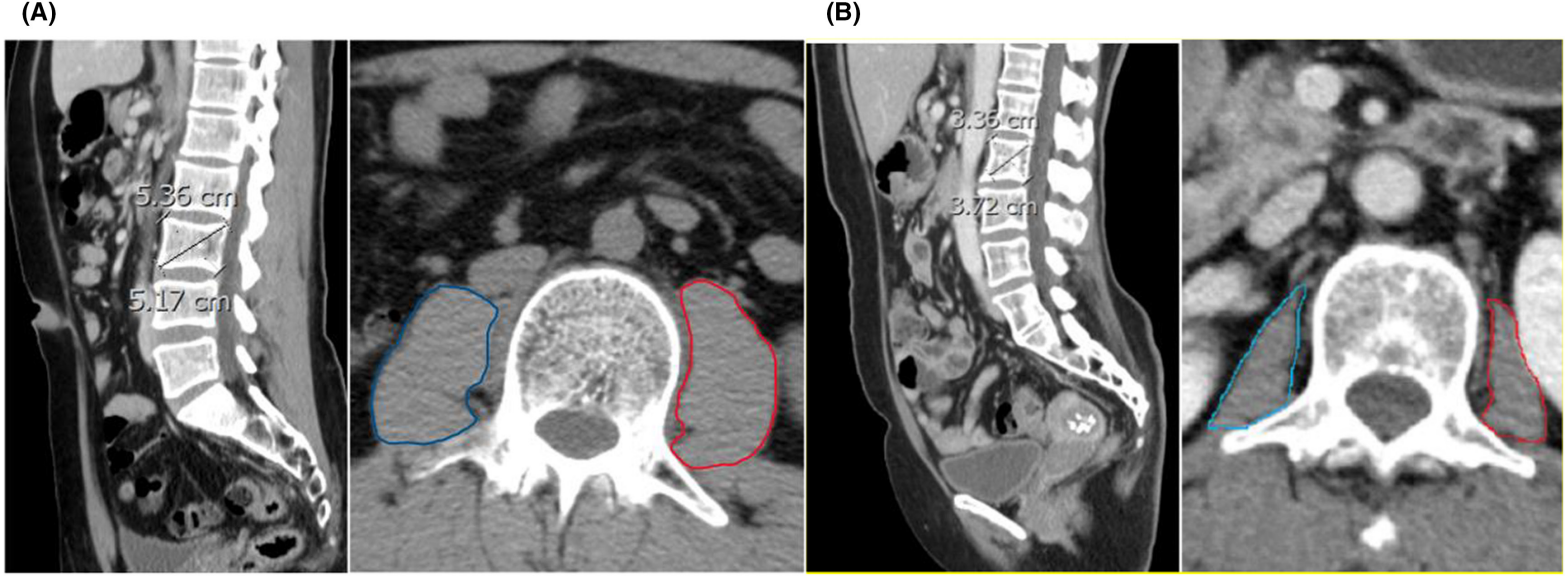
Methodology and technique for psoas measurement. (A) The patient within the third tertile of the psoas muscle area. (B) The patient within the first tertile of the psoas muscle area. The difference in the psoas muscle area is noticeable between (A) and (B).

PMA was often reported as either spine body area‐adjusted PMA (SBA‐PMA) or height‐adjusted PMA (HA‐PMA)^18^, ^20^, ^25^ because these adjusted values take the patient's body habitus into consideration. We divided each of these two metrics into three tertiles (tertile 1: small PMA; tertile 2: medium PMA; tertile 3: large PMA) to allow for clinically meaningful interpretation of odds ratios (OR), the area under the receiver operating characteristic curve (AUC), and the decision curve analysis (DCA).^26^ To account for potential gender differences, the three tertiles of both metrics were calculated independently for males and females. For SBA‐PMA, the tertile ranges were 0 to 0.335, 0.335 to 0.460, and greater than 0.460 (cm^2^/cm^2^) for females, and 0 to 0.456, 0.456 to 0.650, and greater than 0.650 (cm^2^/cm^2^) for males. Similarly, the HA‐PMA tertile ranges were 0 to 0.0185, 0.0185 to 0.0235, and greater than 0.0235 (cm^2^/cm^2^) for females, and 0 to 0.0250, 0.0250 to 0.0350, and greater than 0.0350 (cm^2^/cm^2^) for males. Figure 2A demonstrated a tertile 3 patient with large PMA and Figure 2B demonstrated a tertile 1 patient with small PMA. The Pearson's *r* values of interobserver reliability for the assessment of PMA and SBA were 0.94 and 0.96, respectively.

### 2.6 Baseline characteristics

The clinical and demographic data of our 185 patients are presented in Table 1. The median age of the patients was 62 (interquartile range, 53–70) years old. 48% (89/185) of patients were female. The most common primary tumor sites were the lung (36%, 67/185), breast (13%, 24/185), and liver (11%, 20/185). Survival was 83% (153/185; 95% confidence interval [CI], 0.77–0.88) at 90 days, 43% (71/164; 95% CI, 0.36–0.51) at 1 year, and 24% (37/154; 95% CI, 0.18–0.31) at 2 years. Most baseline characteristics were similar between the three SBA‐PMA tertile groups, except for the platelet count and the absolute neutrophil count. At 90 days, patients with small SBA‐PMA (tertile 1) had a lower probability of survival compared with those with large SBA‐PMA (tertile 3). At 1 year, tertile 1 patients were less likely to be alive than either tertile 2 or tertile 3 patients. The survival probabilities predicted by the three SPAs (PATHFx, SORG‐NG, and SORG‐MLA) were not different between the three tertiles.

**TABLE 1:** Clinicodemographic data of included patients with different tertiles of spine body‐adjusted psoas muscle area (SBA‐PMA).

	Tertile 1 (*n* = 62)	Tertile 2 (*n* = 61)	Tertile 3 (*n* = 62)	*p‐*value
Male	32 (51.6%)	32 (52.5%)	32 (51.6%)	0.994
Median age (IQR)	64 (53–73.25)	63 (55.5–70.5)	58 (50–66)	0.081
Body measurements				
Height	160.7 (7.8)	161.7 (8.3)	161.0 (9.0)	0.905
Weight	59.1 (10.5)	61.0 (11.0)	63.9 (11.2)	0.186
Body mass index	22.1 (4.0)	23.2(3.2)	22.4 (3.2)	0.080
Baseline characteristics				
ASA group	2.8 (0.5)	2.9 (0.5)	3.0 (0.4)	0.192
ECOG (3 and 4)	23 (37.1%)	21 (34.4%)	18 (29.0%)	0.625
Additional comorbidity	42 (67.7%)	34 (55.7%)	36 (58.1%)	0.351
Ambulatory	21 (33.8%)	24 (39.3%)	21 (33.8%)	0.766
Preoperative systemic therapy	39 (65.0%)	41 (68.3%)	43 (71.7%)	0.735
Preoperative radiotherapy	36 (58.0%)	39 (63.9%)	40 (64.5%)	0.715
Laboratory values				
Hemoglobin^a^	12.0 (2.0)	11.2 (1.8)	11.5 (1.9)	0.388
WBC^a^	8.0 (2.8)	6.7 (3.1)	7.8 (3.9)	0.050
Platelet^a^	261.0 (124.2)	219.5 (98.2)	269.4 (127.0)	**0.059**
Tertile 1 versus 2				0.043
Tertile 1 versus 3				0.711
Tertile 2 versus 3				**0.017**
Abs. lym.^a^	17.3 (9.6)	19.0 (10.0)	16.3 (10.0)	0.124
Abs. neutrophil^a^	73.3 (10.7)	69.9 (12.7)	74.7 (11.1)	**0.047**
Creatinine (mg/dL)	0.8 (0.5)	0.9 (1.0)	0.8(0.3)	0.423
Albumin (g/dL)	3.7 (0.6)	3.7 (0.6)	3.7 (0.6)	0.880
ALP (IU/L)	132.3 (119.3)	152.3 (168.4)	141.4 (132.3)	0.844
Sodium	136.1 (3.6)	136.4 (3.1)	136.0 (4.0)	0.837
Calcium	2.3 (0.2)	2.3 (0.3)	2.3 (0.2)	0.878
BUN	18.0 (5.4)	18.3 (6.4)	18.3 (10.9)	0.261
INR	1.0 (0.1)	1.1 (0.5)	1.0 (0.1)	0.402
Oncological status				
Primary tumor type				
Lung (%)		67 (36%)		
Liver (%)		20 (11%)		
Breast (%)		24 (13%)		
Kidney (%)		13 (7%)		
Hematopoietic (%)		8 (4%)		
Prostate (%)		8 (4%)		
Urothelial (%)		7 (4%)		
Colorectal (%)		6 (3%)		
Esophageal (%)		6 (3%)		
Head and neck (%)		5 (3%)		
Others (%)		21 (11%)		
Number of bone metastases				0.423
1 site	14 (22.6%)	11 (18.0%)	18 (29.0%)	
2 sites	3 (4.8%)	7 (11.5%)	4 (6.5%)	
≧3 sites	45 (72.6%)	43 (70.5%)	40 (66.7%)	
Visceral metastases	38 (61.3%)	31 (50.8%)	31 (50.0%)	0.373
Brain metastases	10 (16.1%)	7 (11.5%)	11 (17.7%)	0.603
Survival rate				
90‐day survival	46 (75.4%)	50 (82.0%)	57 (91.9%)	**0.047**
Tertile 1 versus 2				0.381
Tertile 1 versus 3				**0.013**
Tertile 2 versus 3				0.102
1‐year survival	15 (26.8%)	26 (49.1%)	30 (54.6%)	**0.007**
Tertile 1 versus 2				**0.016**
Tertile 1 versus 3				**0.003**
Tertile 2 versus 3				0.572
SPA				
PATHFx				
90‐day PATHFx	0.51 (0.23)	0.52 (0.22)	0.52 (0.23)	0.914
1‐year PATHFx	0.32 (0.21)	0.32 (0.19)	0.34 (0.20)	0.748
SSG score				0.887
A	9 (14.5%)	9 (14.8%)	12 (19.4%)	
B	41 (66.1%)	40 (65.6%)	41 (66.1%)	
C	12 (19.4%)	12 (19.7%)	9 (14.5%)	
SORG‐CA	5.6 (1.8)	5.2 (1.8)	5.0 (1.9)	0.145
SORG‐NG				
90‐day SORG‐NG	0.62 (0.15)	0.66 (0.14)	0.68 (0.15)	0.060
1‐year SORG‐NG	0.29 (0.17)	0.34 (0.17)	0.37 (0.19)	0.060
Tertile 1 versus 2				0.404
Tertile 1 versus 3				0.560
Tertile 2 versus 3				0.339
OPTIModel				0.141
A	2 (3.2%)	8 (13.1%)	8 (12.9%)	
B	13 (21.0%)	5 (8.2%)	6 (9.7%)	
C	27 (43.5%)	26 (42.6%)	23 (37.1%)	
D	20 (32.3%)	22 (36.1%)	25 (40.3%)	
SPRING‐NG				
90‐day SPRING	0.15 (0.13)	0.13 (0.16)	0.11 (0.18)	0.552
1‐year SPRING	0.43 (0.29)	0.42 (0.30)	0.40 (0.30)	0.676
SORG‐MLA				
90‐day SORG‐MLA	0.71(0.27)	0.74 (0.29)	0.72 (0.25)	0.349
1‐year SORG‐MLA	0.31 (0.22)	0.35 (0.26)	0.34 (0.26)	0.903
MEP score	1.4 (0.64)	1.4 (0.70)	1.5 (0.67)	0.863

*Note*: Bold *p*‐values indicate statistical significance (*p* < 0.05). Tertile 1, patients with the smallest spine body‐adjusted psoas muscle area; Tertile 2, patients with the intermediate spine body‐adjusted psoas muscle area; Tertile 3, patients with the largest spine body‐adjusted psoas muscle area.

Abbreviations: Abs., absolute; ALP, alkaline phosphatase; Ambulatory, normal ambulatory status; Charlson, Charlson comorbidity other than cancer or metastases disease; ASA, American Society of Anesthesiologists score; BUN, blood urea nitrogen; Cre., creatinine; INR, international normalized ratio; IQR, interquartile range; lym., lymphocyte; MEP score, metastatic early prognostic score; No., number; SORG, Skeletal Oncology Research Group; SORG‐CA, SORG classical algorithm; SORG‐MLA, SORG machine learning algorithm; SORG‐NG, SORG nomogram; SPA, survival prediction algorithms; SPRING‐NG, SPRING nomogram; SSG, Scandinavian Sarcoma Group; WBC, white blood cell.

^a^ In the unit of (10^3^/μL).

### 2.7 Statistical analysis

Binary clinical and demographic data were compared with chi‐square tests and Yates' correction (if applicable). Continuous variables that passed the normality test were compared with an analysis of variance. Otherwise, they were compared with Kruskal–Wallis test. We used logistic regression to determine the effect of SBA‐PMA or HA‐PMA on postoperative 90‐day and 1‐year survival. Cox proportional hazards regression analysis was conducted to estimate the hazard ratio of overall mortality. Kaplan–Meier survival curves of patients in each SBA‐PMA or HA‐PMA tertile were plotted and compared by log‐rank test. To eliminate the influence of outliers, all data were censored at the patient's death or 2 years after surgery.

The discriminatory abilities of the three SPAs for 90‐day and 1‐year survival predictions were assessed by the AUC (Model 1 in Table 2), which typically ranges from 0.5 to 1.0. An AUC of 1.0 indicates perfect discrimination and 0.5 indicates random guessing. An AUC greater than 0.7 is usually considered an indicator of good discrimination.^27^ Logistic regression analysis was used to evaluate whether adding SBA‐PMA or HA‐PMA into the three SPAs (i.e., Model 2 and 3 in Table 2) influenced the discriminatory ability of their 90‐day and 1‐year predictions.

**TABLE 2:** AUC with 95% confidence intervals of three SPAs (Model 1) incorporating SBA‐PMA (Model 2) or HA‐PMA (Model 3) in logistic regression.

	PATHFx	SORG‐NG	SORG‐MLA
90‐day			
Model 1	0.64 (0.54–0.74)	0.67 (0.57–0.77)	0.72 (0.61–0.83)
Model 2	0.69 (0.59–0.79)	0.72 (0.63–0.81)	0.79 (0.71–0.87)
Model 3	0.70 (0.60–0.80)	0.72 (0.63–0.81)	0.79 (0.71–0.88)
1‐year			
Model 1	0.66 (0.58–0.75)	0.73 (0.65–0.81)	0.78 (0.70–0.85)
Model 2	0.70 (0.62–0.78)	0.74 (0.66–0.82)	0.81 (0.74–0.88)
Model 3	0.69 (0.60–0.77)	0.74 (0.66–0.82)	0.81 (0.73–0.88)

*Note*: No significant AUC changes were observed between the three models. Model 1, simple logistic model comprised of only the preoperative scoring system; Model 2, complex logistic model consisted of the preoperative scoring system plus spine body‐adjusted psoas muscle area (SBA‐PMA); Model 3, complex logistic model composed of the preoperative scoring system and height‐adjusted psoas muscle area (HA‐PMA).

Decision curve analysis evaluates a model's clinical utility by plotting the net benefit across all possible risk thresholds.^26^ The patient and the treating physician can agree on an acceptable risk threshold and infer what the predicted benefit would be. The acceptable threshold probability should ideally be individualized based on the clinical scenario. For example, when prescribing non‐steroidal anti‐inflammatory agents for a young woman with acute tenosynovitis, the clinician may choose a lower threshold probability because the treatment's risk is low. In contrast, a higher threshold probability should be adopted when performing major surgery on a frail patient with multiple comorbidities.

We performed three analyses to assess the influence of PMA on 90‐day and 1‐year survival. The primary analysis was whether a small SBA‐PMA or HA‐PMA was associated with higher rates of postoperative 90‐day or 1‐year mortality in logistic regression analysis. The secondary analysis attempted to determine whether incorporating SBA‐PMA/HA‐PMA into the three SPAs improved their discrimination as measured by AUC. The tertiary analysis was using DCA to assess if the incorporation of SBA‐PMA/HA‐PMA led to increased net benefits. All statistical analyses were performed using R (version 4.0.4). A two‐tailed *p*‐value of <0.05 was considered significant. For the three comparisons in the post hoc analysis, the Bonferroni correction was applied to neutralize the effect of repeated testing. The *p‐*value threshold for statistical significance was thus adjusted to be 0.017.

## 3 RESULTS

### 3.1 Do patients with small PMA on CT have worse 90‐day, 1‐year, and overall survival after undergoing surgery for an extremity metastasis?

**FIGURE 3:**
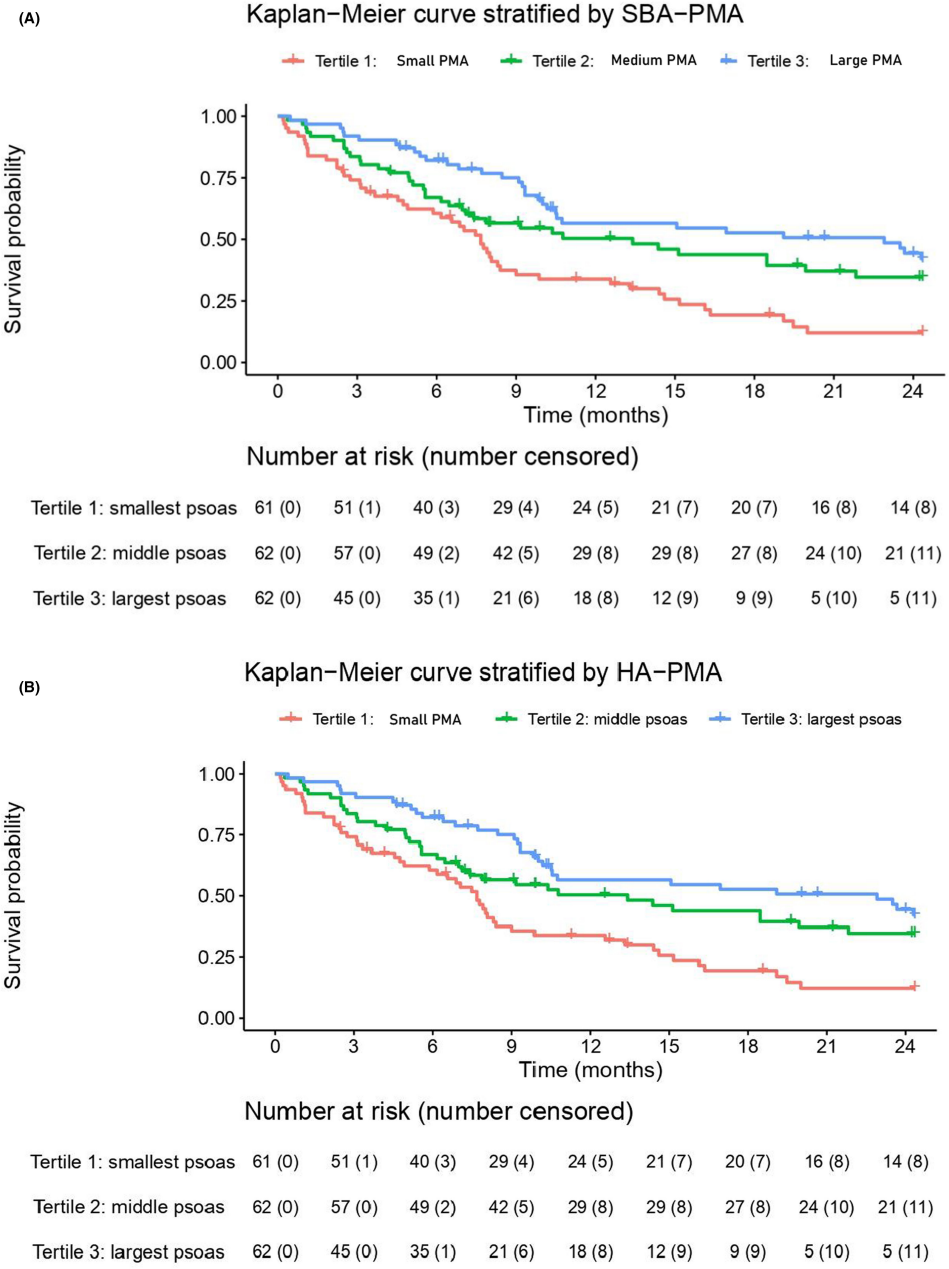
Kaplan–Meier curve for overall survival. The patients were stratified into (A) three tertiles by spine body‐adjusted psoas muscle area (SBA‐PMA) and (B) three tertiles by height‐adjusted psoas muscle area (HA‐PMA). The log‐rank test was significant on both occasions with a *p*‐value smaller than 0.001.

Kaplan–Meier curves demonstrated differences in cumulated survival when patients were stratified by the three tertiles of SBA‐PMA and HA‐PMA (Figure 3). In general, patients with larger PMA had improved survival than patients with smaller PMA (log‐rank test *p*‐value <0.001 in analysis stratified by either SBA‐PMA or HA‐PMA). In logistic regression analysis, tertile 3 patients (large PMA) had a better chance of surviving 90 days than tertile 1 patients, with an OR of 3.72 (95% CI, 1.33–12.13; *p* = 0.02) when stratified by SBA‐PMA and an OR of 4.05 (95% CI, 1.46–13.16; *p* = 0.01) when stratified by HA‐PMA. This survival difference between tertile 3 and tertile 1 patients remained even after adjusting for predictions made by the three SPAs (Table 3). No survival differences were observed between other comparison pairs (i.e., tertile 1 vs. 2 or tertile 2 vs. 3). Compared with patients with small PMA, patients with large PMA had a higher chance of surviving at 1 year, with an OR of 3.28 (95% CI, 1.50–7.41; *p* = 0.004) when stratified by SBA‐PMA and an OR of 2.51 (95% CI, 1.17–5.53; *p* = 0.020) when stratified by HA‐PMA. This 1‐year survival advantage in patients with larger PMA also persisted after controlling for predictions made by the three SPAs. In Cox proportional‐hazard regression analysis (Table 4), tertile 2 and tertile 3 patients had better overall survival compared with tertile 1 patients (SBA‐PMA: T2 vs. T1 HR, 0.52; 95% CI, 0.33–0.80; *p* = 0.004; T3 vs. T1 HR, 0.39; 95% CI, 0.25–0.61; *p* < 0.001; HA‐PMA: T2 vs. T1 HR, 0.57; 95% CI, 0.37–0.87; *p* = 0.010; T3 vs. T1 HR, 0.40; 95% CI, 0.26–0.63; *p* < 0.001). This trend of survival advantage was still observed after controlling for the three SPAs’ predictions.

**TABLE 3:** Odds ratio with 95% confidence intervals for 90‐day and 1‐year survival before and after adjusted for three survival prediction algorithms (PATHFx, SORG‐NG, SORG‐MLA).

	Unadjusted OR	OR adj. PATHFx	OR adj. SORG‐NG	OR adj. SORG‐MLA
SBA‐PMA				
90‐day survival				
T2 versus T1	1.48 (0.62–3.63)	1.49 (0.61–3.72)	1.28 (0.52–3.21)	1.46 (0.59–3.72)
T3 versus T1	**3.72 (1.33–12.13)**	**3.86 (1.36–12.80)**	**3.15 (1.09–10.46)**	**3.84 (1.33–12.89)**
T3 versus T2	2.51 (0.85–8.41)	2.59 (0.86–8.81)	2.46 (0.82–8.42)	2.63 (0.86–9.10)
1‐year survival				
T2 versus T1	**2.63 (1.20–5.96)**	**2.93 (1.28–6.97)**	2.28 (0.98–5.44)	**3.14 (1.25–8.32)**
T3 versus T1	**3.28 (1.50–7.41)**	**3.47 (1.53–8.23)**	**2.46 (1.06–5.87)**	**4.48 (1.79–11.92)**
T3 versus T2	1.25 (0.59–2.67)	1.19 (0.54–2.63)	1.08 (0.47–2.45)	1.43 (0.59–3.49)
HA‐PMA				
90‐day survival				
T2 versus T1	1.81 (0.76–4.52)	1.98 (0.81–5.07)	2.16 (0.87–5.65)	**2.64 (1.02–7.39)**
T3 versus T1	**4.05 (1.46–13.16)**	**4.62 (1.63–15.39)**	**4.06 (1.43–13.50)**	**5.39 (1.84–18.52)**
T3 versus T2	2.24 (0.74–7.58)	2.34 (0.76–8.06)	1.88 (0.60–6.54)	2.04 (0.64–7.18)
1‐year survival				
T2 versus T1	1.79 (0.83–3.94)	1.98 (0.88–4.54)	2.07 (0.90–4.91)	**2.61 (1.04–6.90)**
T3 versus T1	**2.51 (1.17–5.53)**	**2.70 (1.21–6.20)**	2.26 (0.99–5.29)	**4.14 (1.66–10.96)**
T3 versus T2	1.40 (0.66–3.02)	1.37 (0.62–3.06)	1.09 (0.47–2.51)	1.58 (0.65–3.91)

*Note*: Bold indicates significant odds ratios (*p* < 0.05).

Abbreviations: adj, adjusted; HA‐PMA, height‐adjusted psoas muscle area; OR, odds ratio; SBA‐PMA, spine body‐adjusted psoas muscle area.

**TABLE 4:** Hazard ratio with 95% confidence intervals of overall survival unadjusted and adjusted for three survival prediction algorithms (PATHFx, SORG‐NG, SORG‐MLA).

	Unadjusted HR	HR adj. PATHFx	HR adj. SORG‐NG	HR adj. SORG‐MLA
SBA‐PMA				
T2 versus T1	**0.52 (0.33–0.80)**	**0.52 (0.34–0.81)**	**0.63 (0.40–0.98)**	**0.58 (0.37–0.89)**
T3 versus T1	**0.39 (0.25–0.61)**	**0.40 (0.25–0.62)**	**0.50 (0.31–0.79)**	**0.40 (0.25–0.62)**
T3 versus T2	0.76 (0.47–1.22)	0.76 (0.47–1.22)	0.79 (0.49–1.27)	0.69 (0.43–1.10)
HA‐PMA				
T2 versus T1	**0.57 (0.37–0.87)**	**0.58 (0.37–0.89)**	**0.54 (0.35–0.84)**	**0.51 (0.33–0.80)**
T3 versus T1	**0.40 (0.26–0.63)**	**0.39 (0.25–0.62)**	**0.44 (0.28–0.69)**	**0.36 (0.23–0.56)**
T3 versus T2	0.71 (0.44–1.14)	0.68 (0.42–1.10)	0.81 (0.50–1.31)	0.69 (0.43–1.12)

*Note*: Bold indicates significant hazard ratios (*p* < 0.05).

Abbreviations: adj, adjusted; HA‐PMA, height‐adjusted psoas muscle area; HR, hazard ratio; SBA‐PMA, spine body‐adjusted psoas muscle area.

### 3.2 Does incorporating PMA into the three modern SPAs improve their performance?

All SPAs had increased AUC after the incorporation of SBA‐PMA or HA‐PMA (Table 2), although statistical significance was not reached. Logistic regression analysis and Cox proportional hazards regression analysis both showed that small psoas muscle area is associated with decreased survival, even after controlling for estimations made by the three SPAs (Tables 3 and 4).

**FIGURE 4:**
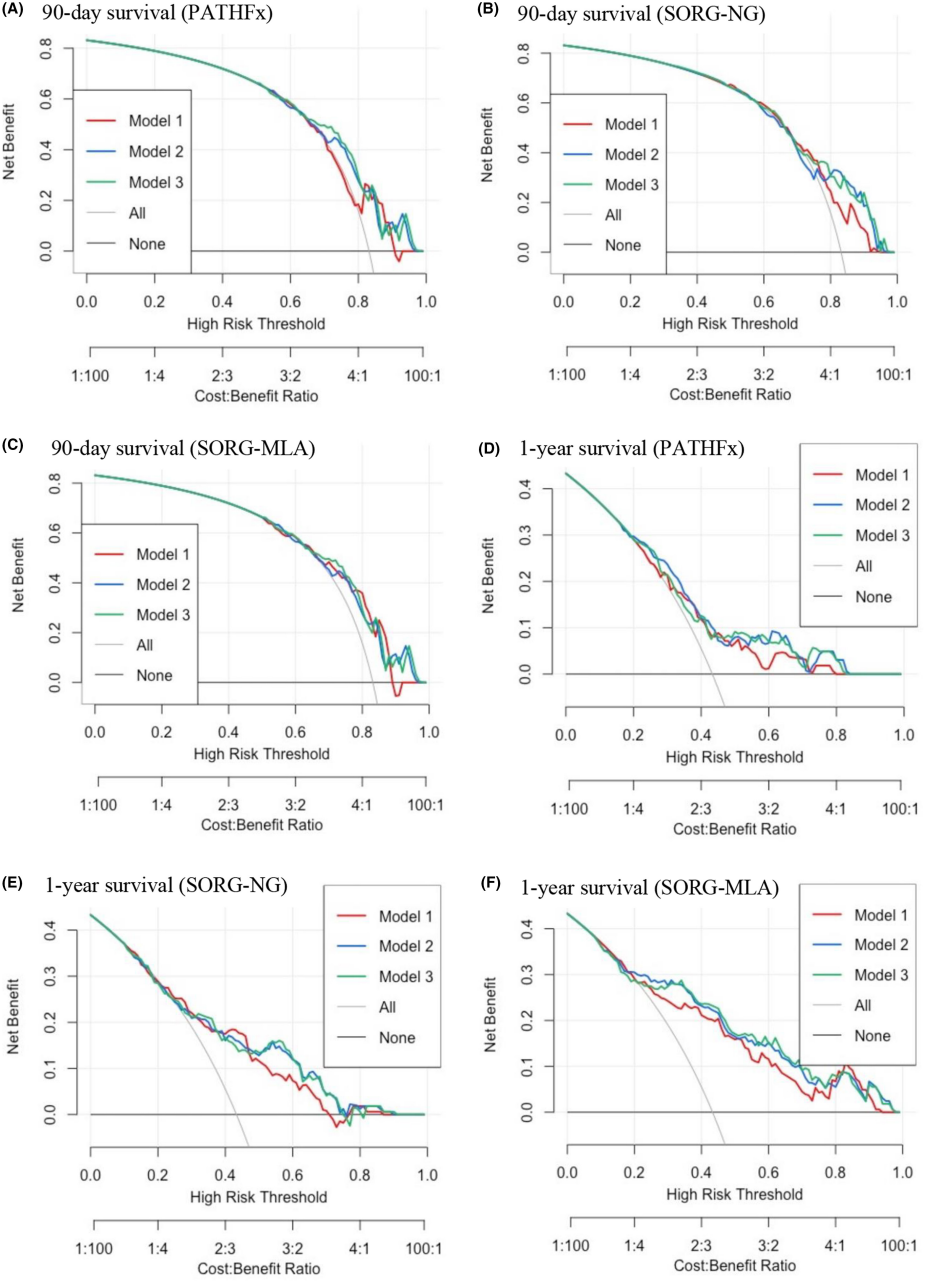
Comparing the 90‐day and 1‐year survival predictions made by “simple” models and their corresponding “complex” models on decision curve analysis. Model 1, simple logistic model comprised of only the preoperative scoring system; Model 2, complex logistic model consisting of the preoperative scoring system plus spine body‐adjusted psoas muscle area (SBA‐PMA); Model 3, complex logistic model composed of the preoperative scoring system and height‐adjusted psoas muscle area (HA‐PMA). PATHFx (A), SORG‐NG (B), and SORG‐MLA (C) predicting 90‐day survival. PATHFx (D), SORG‐NG (E), and SORG‐MLA (F) predicting 1‐year survival.

On decision curve analysis, consulting the 3‐month survival predictions made by PATHFx, SORG‐NG, and SORG‐MLA generally provided net benefits over the default strategy of operating on all patients, especially when the risk thresholds were higher than 0.7 (Figure 4A–C). The addition of SBA‐PMA/HA‐PMA to PATHFx, SORG‐NG, and SORG‐MLA resulted in increased net benefits to these SPAs' 3‐month predictions at risk thresholds ranging from 0.7 to 0.8, 0.8 to 1.0, and 0.9 to 0.95, respectively. The 12‐month predictions of PATHFx, SORG‐NG, and SORG‐MLA conferred net benefits across a wide range of risk thresholds from as low as 0.2 (Figure 4D–F). The increase in benefits was much more pronounced compared to the increase provided by these three SPAs' 3‐month predictions. Adding SBA‐PMA/HA‐PMA to PATHFx, SORG‐NG, and SORG‐MLA's 12‐month predictions imparted more net benefits when the risk thresholds were between 0.5 and 0.7.

## 4 DISCUSSION

Long‐bone metastases are common in patients with advanced cancer and the treatment needs to be individualized. The decision on whether to provide surgery, and on what type of surgery to perform, can be clinically challenging given the complexity of disease and physical status in patients with metastatic cancer. A reliable survival estimate can help clinicians make a more informed decision that maximizes the likelihood of achieving therapeutic benefit and minimizes the chance of unwanted complications. Several survival prediction tools have been developed for this purpose,^15^ but so far none of them incorporated body composition attributes,^5^, ^6^, ^7^, ^8^, ^9^, ^11^, ^12^, ^15^ which could influence survival in patients with cancer.^19^, ^20^, ^25^ Our data showed that patients with a small SBA‐PMA or HA‐PMA had decreased 90‐day, 1‐year, and overall survival, even after controlling for the predictions made by three well‐known survival estimation tools (PATHFx, SORG‐NG, SORG‐MLA). Incorporating either SBA‐PMA or HA‐PMA into these SPAs generally enhanced their discriminatory ability, although statistical significance was not reached. On decision curve analysis, incorporating SBA‐PMA or HA‐PMA into these three SPAs showed increased net benefit typically at higher ends of risk thresholds beyond 0.5. This implied that PMA might have less impact on survival when the treatment was low risk but could play a bigger prognostic role when more extensive, higher‐risk operations were undertaken.

### 4.1 Is decreased PMA on CT associated with worse 90‐day, 1‐year, and overall survival in patients who underwent surgery for an extremity metastasis?

Our study demonstrated PMA could be a survival prognosticator in patients with skeletal metastases in the extremities. Patients with small PMA had lower 90‐day, 1‐year, and overall survival on Kaplan–Meier analysis. Similarly, they had a higher probability of dying at these timepoints on logistic regression analysis. Our findings align with trends observed in previous studies exploring the survival outcomes of patients undergoing surgical treatment for metastases affecting the spine or viscera.^17^, ^18^, ^19^, ^20^, ^21^, ^22^, ^23^, ^25^ These observations were consistent with a recent study by Groot et al. that found decreased PMA on CT was associated with a higher likelihood of mortality at 1 year (Hazard ratio, HR, 1.68; 95% CI, 1.08–2.61; *p* = 0.02) in 212 patients undergoing surgery for extremity metastases.^28^ Although the authors concluded body composition measurements could be used as a novel imaging biomarker to supplement current survival prediction tools for patients with long‐bone metastases, they did not further analyze if predictions made by these tools could actually be improved when morphometric factors were considered. Our study demonstrated that decreased PMA was still associated with higher 90‐day and 1‐year mortality after controlling for the predictions made by three extensively validated SPAs, implying that body composition metrics might capture some prognostic value not currently appreciated by these algorithms. Psoas muscles are involved in various physical tasks such as standing, bending forward, and lifting weights. A healthy size of the psoas muscle might therefore reflect a more active lifestyle^29^, ^30^ and a less severe disease status in patients with extremity metastases. Psoas muscle area (PMA) can be easily measured on CT, is objective, and often remains consistent within a few months. It is also an indicator of muscle wasting syndrome, which commonly affects patients with advanced‐stage cancer and leads to decreased quality of life and poor prognosis.^31^ It is possible that body PMA could capture the patient's physical function, nutritional status, and severity of cancer to some degree in one metric.

### 4.2 Does the incorporation of PMA into existing SPAs improve their performance?

In 2020, the American Musculoskeletal Tumor Society (MSTS), American Society for Radiation Oncology (ASTRO), and American Society of Clinical Oncology (ASCO) jointly published a clinical practice guideline for the treatment of metastatic carcinoma and myeloma of the femur.^32^ In this guideline, the expert panel recommended surgeons “utilize a validated method of estimating survival of the patient in choosing the method of reconstruction.” We tested if the performance of several commonly used survival prediction algorithms could be enhanced with additional consideration of the PMA. Although the discriminatory ability, as indicated by the AUC metric, typically increased when PMA was considered along with the estimations made by these tools, the improvement was not statistically significant. We therefore attempted to investigate if there was any potential benefit of considering PMA besides survival prediction tools by employing decision curve analysis (DCA).^26^ We found that discernible increases in net clinical benefit after the addition of PMA to the three repeatedly validated tools (PATHFx, SORG‐NG, SORG‐MLA) were only observed when the proposed treatment modality had a risk‐to‐benefit ratio >0.7 on 3‐month DCA and >0.5 on 1‐year DCA. These DCA findings suggest that PMA might be more likely to influence patient survival when the proposed treatment carries higher inherent risks such as extensive surgery to resect tumor and achieve durable prosthetic reconstruction. In short, it might be prudent to evaluate patients' PMA when a larger‐scale operation is warranted to address a problematic bone metastasis not amenable to non‐operative treatment or minimally invasive surgery.

### 4.3 Limitations

Our results need to be interpreted with several limitations in mind. Firstly, the study was conducted retrospectively with a relatively small group of patients who had an abdominal CT within 3 months of their index surgery for a long‐bone metastasis. This resulted in the inclusion of only 185 out of the 397 patients initially identified in our databank. Patients who had an abdominal CT often had a primary tumor of intra‐abdominal origin or more extensive disease status manifested as liver and/or spinal metastasis. It is therefore reasonable to assume our study cohort is inherently less healthy and not representative of the entire patient population with extremity metastases. Secondly, we arbitrarily divided SBA‐PMA and HA‐PMA into three tertiles to perform statistical analyses. We could not confidently conclude these PMA cutoff values are readily generalizable to other patient populations. Cutoff values of L3 psoas muscle have been proposed, but they remain controversial.^33^, ^34^, ^35^, ^36^ Asian patients often have smaller body sizes, a higher percentage of adipose tissue, and less muscle mass compared with Caucasians.^37^, ^38^ We believe our PMA cutoff values might be lower than those of the Western counterparts.^33^, ^34^, ^35^ Future studies are needed to identify and validate the best cutoff values of the psoas muscle area for survival prediction in patients with extremity metastases. Thirdly, the effect of incorporating HA‐PMA/SBA‐PMA into some modern, machine learning‐based survival prediction tools, including PATHFx and SORG‐MLA, should best be gauged by comparing their model performance before and after retraining with body morphometric measurements. However, we did not have access to the algorithmic inner workings of PATHFx and SORG‐MLA because we were not involved in the development process. That being said, we feel developers of PATHFx and SORG‐MLA could look into the prognostic potential of body composition factors when they refine and upgrade their algorithms. Lastly. Our findings came from analysis of surgically treated patients with long‐bone metastasis. In clinical practice, the majority of patients with symptomatic bone metastasis likely are initially given radiotherapy if there is no imminent risk of fracture. Readers should be cautioned against directly applying this study's results onto patients who are not surgically treated.

## 5 CONCLUSIONS

In conclusion, this study revealed that decreased psoas muscle area measured on CT is a risk factor for mortality in patients with extremity metastases, even after controlling for three widely known survival prediction tools. Further analysis using DCA demonstrated the added value of considering PMA besides survival prediction tools when clinicians and their patients are contemplating a surgical option of higher magnitude and greater risk. Physicians might want to assess their patients' PMA and counsel them on the related risks when the clinical scenario calls for more extensive surgery to address problematic bone metastasis.

## AUTHOR CONTRIBUTIONS

**Chia‐Che Lee:** Conceptualization (equal); data curation (equal); formal analysis (equal); investigation (equal); methodology (equal); project administration (equal); resources (equal); supervision (equal); validation (equal); writing – original draft (equal); writing – review and editing (equal). **Ting‐En Tseng:** Data curation (equal); investigation (equal); methodology (equal); writing – original draft (equal); writing – review and editing (equal). **Ruey‐Feng Chang:** Conceptualization (equal); project administration (equal); writing – original draft (equal); writing – review and editing (equal). **Hung‐Kuan Yen:** Conceptualization (equal); formal analysis (equal); software (equal); visualization (equal); writing – original draft (equal); writing – review and editing (equal). **Yu‐An Chen:** Data curation (equal); visualization (equal); writing – original draft (equal); writing – review and editing (equal). **Yu‐Yung Chen:** Data curation (equal); writing – original draft (equal); writing – review and editing (equal). **Chih‐Horng Wu:** Data curation (equal); writing – original draft (equal); writing – review and editing (equal). **Ming‐Hsiao Hu:** Data curation (equal); writing – original draft (equal); writing – review and editing (equal). **Mao‐Hsu Yen:** Formal analysis (equal); software (equal); writing – original draft (equal); writing – review and editing (equal). **Michiel Bongers:** Formal analysis (equal); writing – original draft (equal); writing – review and editing (equal). **Olivier Q. Groot:** Formal analysis (equal); writing – original draft (equal); writing – review and editing (equal). **Cheng‐Yo Lai:** Data curation (equal); writing – original draft (equal); writing – review and editing (equal). **Wei‐Hsin Lin:** Conceptualization (equal); data curation (equal); formal analysis (equal); methodology (equal); project administration (equal); resources (equal); supervision (equal); validation (equal); writing – original draft (equal); writing – review and editing (equal).

## FUNDING INFORMATION

The authors received no financial or material support for the research, authorship, and/or publication of this article.

## CONFLICT OF INTEREST STATEMENT

The authors indicated no financial relationships.

## ETHICS STATEMENT

This study followed the Declaration of Helsinki and the STROBE guidelines.^24^ This study was approved by our research ethics committee (201912022RIND). Informed consent was waived due to its retrospective nature.

## Data Availability

The data that support the findings of this study are available on request from the corresponding author. The data are not publicly available due to privacy or ethical restrictions.
